# DEMENTIA RISK FACTORS PREDICT ALTERED WHITE MATTER MICROSTRUCTURE IN A RURAL AGING COHORT

**DOI:** 10.1093/geroni/igae098.4287

**Published:** 2024-12-31

**Authors:** Andrew Bender, Pavithran Pattiam Giriprakash, Dietmar Cordes, Jessica Caldwell, Justin Miller

**Affiliations:** Cleveland Clinic, Las Vegas, Nevada, United States; Cleveland Clinic, Las Vegas, Nevada, United States; Cleveland Clinic, LAs Vegas, Nevada, United States; Cleveland Clinic, Cleveland Clinic, Nevada, United States; University of Washington, Seattle, Washington, United States

## Abstract

Diffusion-weighted magnetic resonance imaging (dMRI) studies of older adults reveal associations between dementia risk factors and altered cerebral white matter microstructure. However, these findings are based almost exclusively on residents of metropolitan areas. Older residents of rural areas face increased risk for Alzheimer’s disease and related dementias, but there are few neuroimaging studies of dementia risk in this population. The present study investigated associations between dementia risk factors and white matter alterations in rural-dwelling older adults (n=76) without dementia, recruited from the Nevada Exploratory Alzheimer’s Disease Research Center. We applied voxel-based analysis (VBA) to separately evaluate established risk factors (APOE e4 carriage, family history of dementia, body mass index [BMI], and diagnosed hypertension, hypercholesterolemia, and sleep apnea) as predictors of differences in dMRI measures of white matter microstructure (fiber density, crossing fiber entropy, fractional anisotropy, and mean diffusivity), while accounting for differences in age, sex, and handedness. VBA results (familywise error corrected p<.05) showed APOE e4 allele carriage, family history of dementia, higher BMI, and self-reported diagnoses of hypertension and sleep apnea are independently associated with widespread patterns of lower white matter fiber density. Moreover, we observed similar patterns of results in subsidiary analyses that also accounted for diagnosed mild cognitive impairment. These are the first findings to characterize associations between elevated dementia risk factors and altered white matter microstructure in rural-dwelling older adults. Future studies should evaluate whether the effects of elevated risk factors on cerebral microstructure differ between rural- and metropolitan-dwelling older adults.

